# The Failure in the Stabilization of Glioblastoma-Derived Cell Lines: Spontaneous *In Vitro* Senescence as the Main Culprit

**DOI:** 10.1371/journal.pone.0087136

**Published:** 2014-01-30

**Authors:** Ewelina Stoczynska-Fidelus, Sylwester Piaskowski, Michal Bienkowski, Mateusz Banaszczyk, Krystyna Hulas-Bigoszewska, Marta Winiecka-Klimek, Anna Radomiak-Zaluska, Waldemar Och, Maciej Borowiec, Jolanta Zieba, Cezary Treda, Piotr Rieske

**Affiliations:** 1 Department of Tumor Biology, Medical University of Lodz, Lodz, Poland; 2 Department of Molecular Pathology and Neuropathology, Chair of Oncology, Medical University of Lodz, Lodz, Poland; 3 Neurological Surgery, The Maria Sklodowska-Curie Regional Specialist Hospital in Zgierz, Zgierz, Lodz, Poland; 4 Clinical Department of Neurosurgery, The Voivodal Specialistic Hospital in Olsztyn, Olsztyn, Warmia and Masuria, Poland; 5 Department of Paediatrics, Oncology, Haematology and Diabetology, Medical University of Lodz, Lodz, Poland; Virginia Commonwealth University, United States of America

## Abstract

Cell line analysis is an important element of cancer research. Despite the progress in glioblastoma cell culturing, the cells isolated from the majority of specimens cannot be propagated infinitely *in vitro.* The aim of this study was to identify the processes responsible for the stabilization failure. Therefore, we analyzed 56 primary GB cultures, 7 of which were stabilized. Our results indicate that senescence is primarily responsible for the glioblastoma cell line stabilization failure, while mitotic catastrophe and apoptosis play a minor role. Moreover, a new technical approach allowed for a more profound analysis of the senescent cells in primary cultures, including the distinction between tumor and normal cells. In addition, we observed that glioblastoma cells in primary cultures have a varied potential to undergo spontaneous *in vitro* senescence, which is often higher than that of the normal cells infiltrating the tumor. Thus, this is the first report of GB cells in primary cell cultures (including both monolayer and spheroid conditions) rapidly and spontaneously becoming senescent. Intriguingly, our data also suggest that nearly half of GB cell lines have a combination of *TP53* mutation and *CDKN2A* homozygous deletion, which are considered as mutually exclusive in glioblastoma. Moreover, recognition of the mechanisms of senescence and mitotic catastrophe in glioblastoma cells may be a step towards a potential new therapeutic approach.

## Introduction

Cell line analysis is important in various aspects of cancer research, including exploration of the molecular mechanisms, investigation of cancer cell biology and research for new antineoplastic agents. It is well known that the classical *in vitro* conditions (monolayer, medium with 10% serum) do not enable the culturing of many glioblastoma (GB) cells, especially of these with *EGFR* amplification [1]–[5]. Recently, we have shown that cells with *IDH1* mutation are also negatively selected, which further indicates that a successful glioma cell culturing requires a specific concern [6]. A negative *in vitro* selection of GB *versus* normal cells (most likely glioblastoma associated stromal cells, GASCs, a non-neoplastic stromal cell population surrounding and infiltrating the tumor *in vivo*) was also observed [7]. GASCs can be more adaptable to the classical culture conditions for several possible reasons including their better adhesion ability, higher proliferation rate and the lack of spontaneous apoptosis. Therefore, the ratio of these cells to tumor cells would increase with every passage. However, the exact mechanism responsible for the normal *versus* tumor cell preferential adaptation *in vitro* remains elusive.

Lee *et al.* and Pollard *et al.* independently proposed the novel monolayer conditions (serum-free media, bFGF, EGF, laminin coating, accutase) meant to enable glioblastoma cell culturing in a way to preserve their original genotype and phenotype with a special interest in the propagation of the cells with stem cell markers [3], [8]. It is a crucial aspect, as these cells may be critical for the maintaining of the whole glioblastoma cell culture. Pollard *et al.* showed Nestin and SOX2 as characteristics of stem cells. Nevertheless, controversy over glioblastoma stem cells increases. Some authors suggested CD133 as characteristic for glioma stem cells, other have shown that CD133 negative cells can be tumorigenic in SCID mice [9], [10]. In addition, recently CD133 expression has been shown in glioblastoma infiltrating endothelial cells [11].

Moreover, the conditions proposed by Lee *et al.* and Pollard *et al.* still are not adequate for many glioblastoma cells; *e.g.* in the studies by Lee *et al.* the status of the cells with *EGFR* amplification was either presented elusively or not presented at all [3], [8]. On the other hand, in accordance with our previous findings [1], [12] Stockhausen *et al.* showed that such cells may be temporarily maintained by means of 3D cell culture conditions [13].

In comparison to other groups analyzing the stabilized cell lines, we focused on the cases which do not provide the infinitely proliferating cells. The aim of this study was to identify the processes responsible for the failure in the stabilization of glioblastoma cell lines. Recognizing such mechanisms may offer new culture protocols allowing to propagate the majority of GB cells instead of the few selected types. Moreover, the identification of these mechanisms may be followed by a new therapeutic approach – their induction or inhibition *in vivo*.

## Materials and Methods

### Cell Culture

#### Tumor samples

Tissue samples were obtained from 56 patients diagnosed with glioblastoma treated at the Department of Neurological Surgery, The Maria Sklodowska-Curie Regional Specialist Hospital in Zgierz and at the Clinical Department of Neurosurgery, The Voivodal Specialistic Hospital in Olsztyn. In 3 cases the sample originated from a recurrent tumor treated with radiochemotherapy after the first surgery. All samples were collected using the protocol approved by the Bioethical Committee of the Medical University of Lodz (Approval No RNN/9/10/KE). Written informed consent was obtained from all patients and their data were processed and stored according to the principles expressed in the Declaration of Helsinki. The patients were diagnosed according to the World Health Organization Criteria for Brain Tumor Classification (2007).

Irrespective of cell culture type, the isolation of cells from fresh glioblastoma specimens started within 3 hours after neurosurgical operation. Neurosurgical specimens were shipped in 1x Hank’s Balanced Salt Solution (PAA, The Cell Culture Company, Austria).

#### Establishment and growth of GB cells under the classical culture conditions

Fresh glioblastoma samples were washed twice with 1x Hank’s BSS and centrifuged 90 s at 80 x g each time. Then, the sample was transferred to a 10 cm dish, where it was cut into <1 mm^3^ fragments, washed with 1x Hanks’ BSS. Tumor cells were dispersed with collagenase type IV (200 U/mL, 37°C for 6 h; Sigma-Aldrich, USA). The cells were then cultured in αMEM medium (PAA) containing NEAA and supplemented with 10% FBS (Gibco). The total time of isolation and establishment of cell cultures was about seven hours. Depending on the rate of proliferation, the cells were passaged with Trypsin-EDTA (0.05% trypsin; Gibco) to a new culture dish every 7–14 days.

#### Establishment and growth of GB cells in monolayer serum-free conditions

Fresh glioblastoma samples were washed twice with 1x Hank’s BSS and centrifuged 90 s at 80×g each time. Then, the sample was transferred to a 10 cm dish, where it was cut into <1 mm^3^ fragments and washed with 1x Hanks’ BSS. Tumor cells were dispersed with collagenase type IV (200 U/mL, 37°C for 6 h). The total time of isolation and establishment of cell cultures was about seven hours. The cells were cultured in Neurobasal Medium supplemented with N2 and B27 (0.5x each; Invitrogen), human recombinant bFGF (50 ng/mL; Invitrogen), EGF (50 ng/mL; Invitrogen) and NEAA (1x; Gibco). For monolayer cultures, the plates were precoated with a poly-L-lysine/laminin mixture (Invitrogen) as previously reported [3]. Monolayer cells were passaged with accutase (Invitrogen) [8]. The cells under these conditions were passaged less often (every 2–4 weeks) due to their lower proliferation rate.

#### Establishment and growth of GB cells in spheroids

Spheroid cell cultures were performed as neurospheres and as adherent spheres [1], [14], [15]. Fresh glioblastoma samples were washed twice with 1x Hank’s BSS) and centrifuged 90 s at 80×g each time. Then, the sample was transferred to a 10 cm dish, where it was cut into <1 mm^3^ fragments, washed with 1x Hanks’ BSS, digested with collagenase type IV/dispase (200 u/mL; both Sigma-Aldrich) for 30 min at 37°C and then filtered using a 70 µm cell strainer (BD Biosciences, USA). Filtered cells were washed twice with 1x Hank’s BSS and centrifuged 90 s at 80×g each time and then seeded into 6-well plates at 2500–5000 cells/cm^3^. Having prepared the medium and reagents before glioblastoma samples processing, the total time of isolation and establishment of spheroid cell cultures was about one hour. The culture consisted of Neurobasal Medium with B27 supplement (20 µl/mL; Invitrogen), Glutamax (10 µl/mL; Invitrogen), fibroblast growth factor-2 (20 ng/mL; Invitrogen), NEAA and heparin (2 µg/mL; StemCell Technologies). Growth factors and heparin were renewed twice a week. For the adherent sphere conditions Matrigel covered plates were used and medium was not supplemented with heparin. The spheres were split by mechanical dissociation and transferred to new dish when they reached the size of 200–500 µm.

### Cells were Cultured at 37°C in 5% CO_2_, 95% Humidity and without O_2_ Control

#### DNA/RNA isolation and reverse transcriptase PCR

Total cellular DNA and RNA were isolated from frozen tissue samples (stored at −80°C), the corresponding cell cultures and frozen leukocytes from peripheral blood obtained from patients and healthy volunteers using an AllPrep DNA/RNA Mini Kit (Qiagen, Germany) according to the manufacturer’s protocol. RNA and DNA concentrations were measured spectrophotometrically. 100 ng of total RNA was reverse transcribed into a single-stranded cDNA using a QuantiTect Rev. Transcription Kit (Qiagen) according to the manufacturer’s protocol. DNA and RNA were isolated from all samples (n = 56), for which all molecular analyses (Real-time PCR for *EGFR*, *CDKN2A*, *PDGFRA* and *EGFRvIII*, TP53 sequencing and MLPA) were performed. STR analysis gave informative results in 32 samples (n = 32) and FISH analyses as a verification of other *EGFR* analyses was performed for 19 samples (n = 19) including the 7 stabilized cell lines.

### *EGFR* Gene Analysis by Quantitative Real-Time PCR at the DNA Level

For *EGFR* amplification detection the novel method was applied [16]. To determine the *EGFR* gene dosage level in each sample quantitative Real-Time PCR was performed using StepOnePlus™ Real-Time PCR System (Life Technologies). Each sample was amplified in triplicate in a 10 µl reaction volume containing 10 ng of DNA, a 1x reaction mixture containing Syto9 (Life Technologies, US) and 35 ng each of the forward and reverse primers (Tab. S1). The cycling conditions for the Real-Time PCR reactions were as follows: 3 min at 95°C (polymerase activation) followed by 40 cycles of 20 s at 95°C (denaturation), 30 s at 60°C (annealing) and 20 s at 72°C (extension). The gene dissociation curve was analyzed and the normalized relative gene dosage level was calculated using the method described by Pfaffl *et al.* [17]. Real-time PCR efficiency was calculated using LinReg software. DNA derived from peripheral blood leukocytes was used as a control. Chromosome 7 polysomy was identified when the ratio of *GPER* to *RNaseP* was higher than 1.5; while *EGFR* amplification was identified when the ratio of *EGFR* to *GPER* was higher than 1.5. The results obtained from Real-Time PCR were cross-validated with data obtained from MLPA and FISH.

### Analysis of the *EGFRvIII*, *EGFR, NF1, TP53, PDGFB, CHI3L1, MGMT, XRCC1, GABRA1* and *HES1* Expression at the cDNA Level by Quantitative Real-Time PCR

To determine the *EGFRvIII*, *EGFR, NF1, TP53, PDGFB, CHI3L1, MGMT, XRCC1, GABRA1* and *HES1* expression quantitative Real-Time PCR reactions were performed as described above. The *EGFRvIII*-specific primers were based on a previous report [18]. *GUSB* was used as a reference gene for the normalization of the target gene expression level. The primer sequences are listed in table S1. To evaluate the *EGFRvIII* expression, cDNA derived from tumor tissue positive for *EGFRvIII* was used as the control. To normalize the expression of the other mentioned genes, commercial cDNA from normal brain was used as the control. The Real-Time PCR was preceded by a conventional RT-PCR applied to examine the tested tumor samples in terms of expression of *EGFRvIII*.

### Detection of *CDKN2A* Deletions at the DNA Level by Quantitative Real-Time PCR

The presence of *CDKN2A* exon 1 and/or exon 2 deletions in the original tumor tissue and cultured cells was analyzed by quantitative Real-Time PCR using a StepOnePlus™ Real-Time PCR System (Life Technologies). The primer sequences for *CDKN2A* exon1 and exon 2 are listed in table S1. Real-Time PCR reactions were performed as described above. DNA derived from a non-neoplastic tissue (leukocytes) was used as a control in which the gene dosage level was assumed as 1. Each sample was analyzed three times. An average value lower than 0.3 was considered to represent the deletion of the tested gene in the general population, while a value between 0.3 and 0.75 was considered to represent the deletion of the tested gene in a subpopulation(s). *CDKN2A* exon 1 and/or exon 2 deletion was confirmed by an agarose gel electrophoresis using BioRad Quantity One 1-D Analysis Software. The results obtained from Real-Time PCR were cross-validated with data obtained from MLPA.

### Fluorescence *in situ* Hybridization (FISH)

FISH was performed with FISH Pretreatment Reagent Kit (Abbott Molecular, US) according to the manufacturer’s protocol. In brief, a commercial probe set (Vysis LSI EGFR SpectrumOrange/CEP 7 SpectrumGreen; Abbott Molecular, US) was used to simultaneously detect the copy numbers of the *EGFR* gene and of chromosome 7. FISH was performed using the following procedure: the fixed slides were incubated in 2x standard saline citrate (SSC; Sigma-Aldrich) at 72°C for 5 min, immersed in protease solution (Sigma-Aldrich) for 10 min at 37°C, washed with PBS for 5 min at room temperature, fixed with 1% formaldehyde for 5 min at room temperature, washed in PBS for 5 min at room temperature, dehydrated in 70%, 85% and 100% ethanol for 1 min each, then air-dried and placed on a 50°C slide warmer for 2 min. The FISH probe mix was centrifuged and denatured at 73°C for 5 min. The denatured probe was added to each specimen. The slides were then coverslipped and incubated at 37°C overnight in a humidified chamber. Next, the slides were washed with 0.4x SSC/0.3% NP-40 (Sigma-Aldrich) at 73°C for 2 min, rinsed in 2x SSC/0.1% NP-40 for 1 min at room temperature and air dried in darkness. Before coverslipping, 10 µl of DAPI II counterstain (Molecular Probes) was added to the slides. To score the samples, Nikon Eclipse Ci-S fluorescence microscope was used. The number of red signals, caused by the binding of the *EGFR*-specific probe, directly reflects the number of copies of *EGFR*. The number of green signals, caused by the binding of the CEP 7 probe, directly reflects the number of copies of chromosome 7. FISH evaluation was performed using previously published criteria [19]. For each sample at least 100 nuclei were analyzed. The *EGFR*/CEP 7 ratio was calculated and samples containing three or more signals specific for CEP 7 per nucleus were defined as having chromosome 7 polysomy. Samples with intrachromosomal amplification ratios of 2 or greater were considered to be amplified for *EGFR*. Extrachromosomal amplification of *EGFR* was defined as the presence of at least three times as many *EGFR* signals as centromere 7 signals per cell [20].

### *TP53* Sequencing Analysis

The *TP53* gene mutations were analyzed in exons 5 to 8. The primers used for the PCR amplification of cDNA sequences and sequencing primers are listed in table S1. cDNA sequencing was performed using BigDye Seq kit v3.1 (Applied Biosystems, Foster City, CA, USA). The sequences were analyzed with the ABI 3130 genetic analyzer and DNA Sequencing Analysis Software (Applied Biosystems, Foster City, CA, USA).

### Analysis of Short Tandem Repeats (STR)

Short tandem repeats were analyzed in peripheral blood samples, tumor samples and cell cultures derived from these samples. The following STR markers were used: D1S508, D1S510, D1S2734, D9S162, D9S319, D10S587, D10S1267, D13S53, D13S126, D13S256, D13S263, D17S976, D17S1828, D19S206, D19S867, D22S1150, D22S1163. PCR assays were performed using the thermocycling conditions optimised for each pair of primers. PCR products were denatured and gel electrophoresis was conducted with approximately 0.2–0.5 µl of each product using a Li-Cor automated sequencer system (Lincoln, NE, USA). The relative intensity of bands representing two alleles from one locus (%) was assessed densitometrically (using BioRad Quantity One 1-D Analysis Software) with the assumption that the cumulative value is equal to 100%.

### Multiplex Ligation-dependent Probe Amplification (MLPA)

The MLPA reactions were performed using the commercially available probemixes (P175 and P294) and kits (MRC-Holland, Netherlands) according to the manufacturer’s protocol. In brief, 5 µl samples with 50–250 ng of genomic DNA were denatured at 98°C for 5 min and then cooled to 25°C. Next, 3 µl of hybridization mastermix (containing 1.5 µl of MLPA buffer and 1.5 µl of probemix per sample) was added to each sample and incubated at 98°C for 1 min and at 60°C for 16–20 h. Next, without removing the tubes from the thermocycler (paused at 54°C), 32 µl of ligase mastermix (containing 25 µl water, 3 µl ligase buffer A, 3 µl ligase buffer B and 1 µl ligase per sample) was added to each sample and incubated at 54°C for 15 min and at 98°C for 5 min, then cooled to 20°C. Finally, 10 µl of polymerase mastermix (containing 7.5 µl water, 2 µl SALSA PCR primer mix and 0.5 µl polymerase per sample) was added to each sample and the cycling conditions were as follows: 35 cycles of denaturation at 95°C for 30 s, annealing at 60°C for 30 s and elongation at 72°C for 60 s followed by incubation at 72°C for 20 min. The products were cooled to 15°C and stored in a dark box at 4°C. The fragments were separated by capillary electrophoresis using ABI 3130 genetic analyzer (Applied BioSystems). The comparative analyzes were performed using Coffalyzer.Net v130202.2357 (MRC-Holland). The resultant ratio for given gene of more than 1.3 was interpreted as a gain, while of less than 0.7 as a loss. The results for *EGFR* and *CDKN2A* were used as a cross-reference for the results obtained with Real-Time PCR. The results for *PDGFRA* of more than 1.3 were interpreted as an increased gene copy number and were presented in table S2.

### Classification into Molecular Subtypes

The classification has been performed analogously to the method proposed by Le Mercier *et al.* [21], but basing on the genomic data instead of immunohistochemistry. Firstly, the patients with *PDGFRA* amplification and *TP53* mutation were classified as Proneural-like, then, those among the others with *EGFR* amplification were classified as Classical-like (Tab. S2). Additionally, we tried to classify the samples into molecular subtypes proposed by Verhaak *et al.* and Noushmehr *et al.* [22], [23] basing on the expression data, however, without reaching unambiguous conclusions (the calculated ratios presented in table S3).

### Immunocytochemistry

For the immunocytochemical analyses spheroid and monolayer cell cultures were fixed in 4% paraformaldehyde for 10 min in PBS and permeabilized with 0.1% Triton X-100 for 10 minutes at room temperature. Nonspecific binding sites were blocked by incubation with 2% donkey serum (Sigma) in PBS for 1 h. For double or triple immunolabeling, the fixed cells were subsequently incubated with the appropriate primary antibodies (Tab. 1) for 1 h at room temperature. Double or triple labeling was visualized by simultaneous incubation with a combination of species-specific fluorochrome-conjugated secondary antibodies (1 h, room temperature) (Tab. 1). The control samples were incubated with the secondary antibodies alone or with the matched isotype controls instead of the primary antibody and were otherwise processed identically. The slides were mounted with ProLong® Gold Antifade Reagent or ProLong® Gold Antifade Reagent with DAPI (Molecular Probes, Invitrogen), coverslipped and examined using Nikon Eclipse Ci-S fluorescence microscope.

**Table 1:** Primary and secondary antibodies used for immunocytochemical staining.

Primary antibodies used for immunocytochemical staining						
AB I	Host		Manufacturer		Dilution	
anti-EGFR (528)	mouse		Santa Cruz Biotechnology, Inc., sc-120		1∶ 100	
anti-EGFR	rabbit		Millipore, 04-338		1∶ 50	
anti-BrdU	mouse		Sigma-Aldrich, B 8434		1∶ 500	
anti-TP53	rabbit		Santa Cruz Biotechnology, Inc., sc-6243		1∶ 100	
anti-GFAP	mouse		Chemicon, MAB360		1∶ 400	
anti-GFAP	goat		Santa Cruz Biotechnology, Inc., sc-6171		1∶ 50	
anti-GFAP	rabbit		Abcam, ab7260		1∶ 1000	
anti-αSMA	mouse		RD Systems, MAB1420		1∶ 1000	
anti-SOX2	rabbit		Millipore, AB-5603		1∶ 500	
anti-Nestin	Rabbit		Proteintech Europe, 19483-1-AP		1∶ 50	
**Secondary antibodies used for immunocytochemical staining**						
**AB II**		**Host**		**Manufacturer**		**Dilution**
anti-mouse Alexa Fluor®594		donkey		Molecular Probes, Invitrogen		1∶ 500
anti-rabbit Alexa Fluor®488		donkey		Molecular Probes, Invitrogen		1∶ 500
anti-goat Alexa Fluor®350		donkey		Molecular Probes, Invitrogen		1∶ 500

### 5-Bromo-2′-deoxyuridine Incorporation Assay (BrdU Co-staining)

To assess the proliferation of glioblastoma cells, 10 µM BrdU was added to the GB cell cultures. After 48 h–14 days of incubation (time depended on the experiment), the tested cultures were processed for immunocytochemical BrdU co-staining. Firstly, an immunocytochemical staining for other markers was performed as described, up to the step of PBS washing after the incubation with the secondary antibodies (Tab. 1). Next, the cells were post-fixed in 4% paraformaldehyde and permeabilized with 0.1% Triton X-100 for 10 min at room temperature. Nonspecific binding sites were blocked by the incubation with 2% donkey serum in PBS for 30 min. After blocking, the cells were treated with 2N HCl in 37°C for 40 min and then with 0.1 M borate buffer (pH 8.5) at room temperature for 12 min. Then, the cells were incubated with anti-BrdU antibody for 1 h, washed with PBS and incubated with the appropriate secondary antibody at room temperature for 1 h (Tab. 1). Finally, the cells were mounted with ProLong® Gold Antifade Reagent, coverslipped and examined using Nikon Eclipse Ci-S fluorescence microscope. For each analysis 200 nuclei were examined. Proliferation rate was defined as the percentage of BrdU-positive cells both in normal and neoplastic cells after the 7-day incubation period.

### Senescence Associated (SA)-β-Gal Staining

SA-β-Gal staining was performed following the protocol by Dimri *et al.* [24]. Cells were washed three times with PBS and fixed with cold 3% paraformaldehyde for 5 min. The cells were than washed two times with PBS for 5 min. Next, a fresh senescence-associated staining solution (1 mg/mL 5-bromo-4-chloro-3-indolyl β-D-galactopyranoside, X-Gal in dimethylformamide (stock 20 mg/mL)/40 mM citric acid/sodium phosphate, pH 6.0/5 mM potassium ferrocyanide/5 mM potassium ferricyanide/150 mM NaCl/2 mM MgCl2), pre-warmed to 37°C, was added and the cells were incubated in 37°C (no CO_2_) for 12 h. After the incubation, the cells were washed two times with PBS for 5 min and photographed using Olympus CKX41 microscope. The percentage of the stained cells was calculated.

### Combination of Methods to Analyze Senescence

For the purpose of this study we developed a method based on the combination of three different technics (SA-β-Gal staining, immunofluorescence, BrdU incorporation assay) to identify and determine the phenotype of the cells showing the features of senescence at the single cell level. SA-β-Gal activity was recognized as the hallmark of senescence. Lack of BrdU incorporation after 14 days and growth of cell bodies to the minimum size of 150 µm and to the length of 500 µm were recognized as a supportive information. Moreover, in order to verify the rate of GB cell proliferation, in several cases *in vitro* real time microscopy observation was performed using JuLi Smart Fluorescent Live Cell Imager (Bulldog Bio Inc.). The images were performed every 3 hours for 9–14 days depending on a case.

### Invasion Assay

The invasion assay was performed using BD BioCoat Matrigel Invasion Chambers (#354480) according to the manufacturer’s protocol. The aim was to investigate the possible correlation of the invasive potential of GB-derived cells with their level of SA-β-Gal activity. For that purpose the spheroids from 5 different cases (52–56) from early and late transfers were plated on the top well of prehydrated Matrigel invasion chambers at a density of 5 spheroids/well/sample in DMEM supplemented with 0.1% FBS. The bottom wells were filled with DMEM supplemented with 10% FBS. The chambers were placed in an incubator for 72 h for the invasion to take place. Afterwards, the spheroids from the top wells were removed and the filters with invading cells were fixed in 4% paraformaldehyde (Sigma) in PBS for 10 min and stained for SA-β-Gal activity and with DAPI. The removed spheroids were transferred to new culture plates covered with Matrigel and after 24 h of culture were also fixed and stained for SA-β-Gal activity and with DAPI. The cells in the bottom wells were counted in the 5 fields corresponding to the 5 spheroid locations per sample with the distinction for SA-β-Gal activity using a Nikon Eclipse Ci-S fluorescence microscope. The invasion was quantified as the percentage of invading cells from each spheroid (the number of cells in a spheroid was estimated basing on each spheroids diameter and the average diameter of cells in the spheroid) separately for SA-β-Gal-positive and -negative cells.

### Identification of the Cells Undergoing Mitotic Catastrophe or Apoptosis

The morphology of cell nuclei was assessed using DAPI in order to detect the cells undergoing apoptosis or mitotic catastrophe. The identification of the cells undergoing apoptosis was based on the typical apoptotic morphology (the condensation of chromatin followed by the formation of apoptotic bodies). Huge planar bi−/polinucleated cells were assumed to be undergoing mitotic catastrophe [25]. Both phenomena were assessed with a Nikon Eclipse Ci-S fluorescence microscope.

### Comparative and Statistical Analyses

In order to compare the culturing conditions reliably, we marked the cultures as early and late passages taking into account the typical differences between the conditions and the individual characteristics of each culture. Therefore, for the classical conditions early passages represent passages 0 to 3, which corresponds to the first three weeks of the culture. The passages 6 to 13 (8–16 weeks) were considered as late passages. For the serum-free conditions, due to their markedly lower proliferation rates, the early and late passages represent p0-p1 (1–3 weeks) and p3-p5 (6–16 weeks), respectively. In case of the spheroid cultures, the transfers 0 to 2 (up to 3 weeks) were considered as early passages, while the transfers 4 to 8 as late passages. The wider spectrum of the late passages results from the differences in each cultures’ individual characteristics, most importantly, in their proliferation rate. The number of samples for which all cell biology methods (real-time microscopy observation, BrdU incorporation assay, with SA-β-Gal staining and immunocytochemical analyses) were applied was 41 (7 stabilized cell lines and 34 unstabilized cell cultures; both for early and late passages/transfers for each culture type). For the first 15 samples we have obtained only fragmentary data.

The statistical analyses were performed using STATISTICA 10.1 software (StatSoft, US). For the analysis of cell biology characteristics Mann-Whitney U test (with α equal to 0.05) was applied to assess the differences between groups (the analysis of BrdU incorporation refers only to the data obtained from experiments with a 7-day incubation). For the analysis of molecular characteristics Χ^2^ test was used (with α equal to 0.05).

### Database Analysis

The analysis of *TP53, CDKN2A, EGFR* and *PDGFRA* status in human cancer cell lines was performed using The Broad-Novartis Cancer Cell Line Encyclopedia (CCLE), which is a collaborative project between the Broad Institute and the Novartis Institutes for Biomedical Research and its Genomics Institute of the Novartis Research Foundation. This database involved a detailed genetic characterization (DNA copy number, mRNA expression and mutation data) of a large panel of human cancer models included about 1000 cell lines [26]. This database does not contain data on *EGFRvIII* expression in cell lines, therefore, in this aspect we based our analyses on the literature data [27]. The results of molecular analyses for cell lines were compared with the population data [28]–[32].

## Results

### Cell Line Stabilization

**Figure 1:**
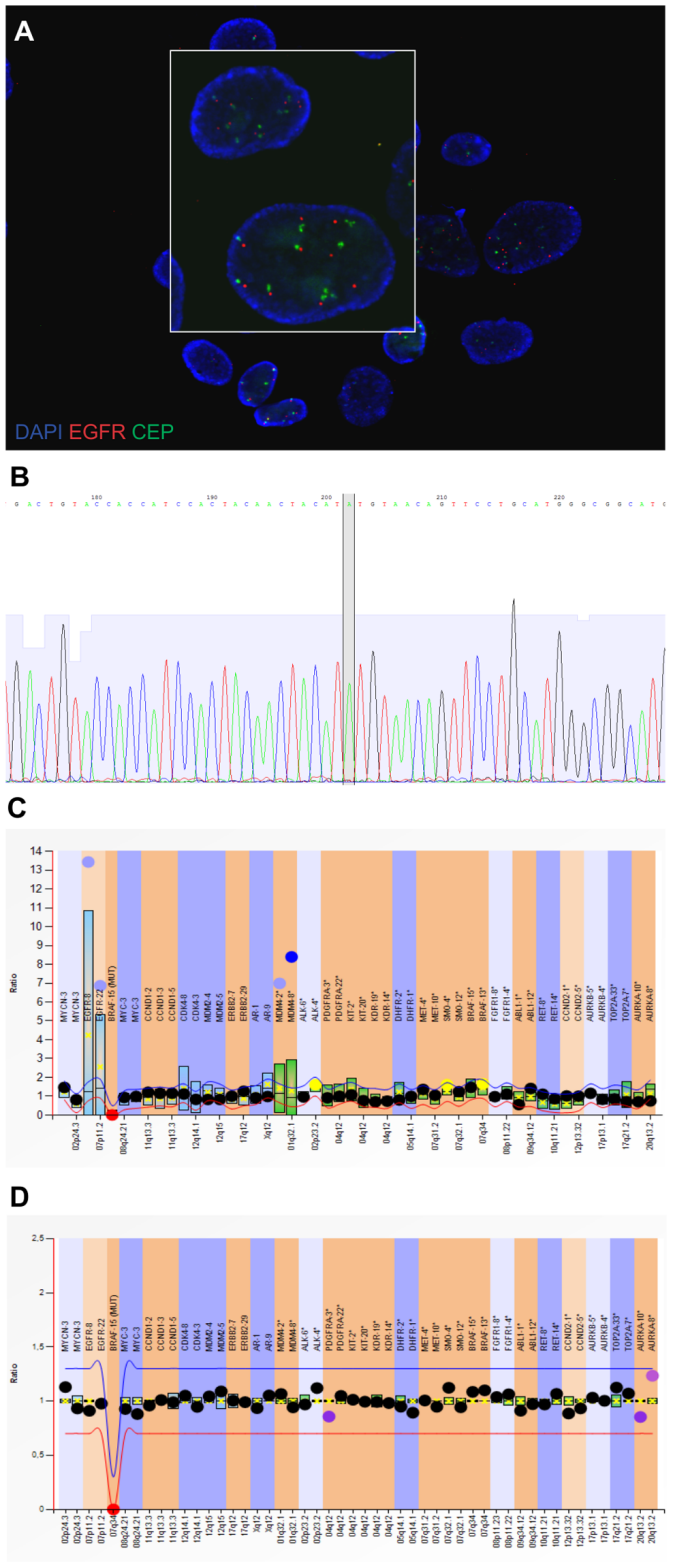
Exemplary results of molecular analyses in glioblastoma cells. (A) An exemplary FISH result presenting chromosome 7 polysomy (a single cell magnified in the rectangle); *EGFR* probe (red signals); CEP 7 control probe (green signals); magnification 1000x; (B) An exemplary *TP53* sequencing result, the line marks the mutated nucleotide in codon 237; (C,D) Exemplary MLPA results showing (C) the genetic alterations (*EGFR* and *MDM4* amplification) in an early passage of cell cultured under classical conditions and (D) their absence in a late passage of the cells from the same tumor.

The cell line stabilization was successful in 7 of the 56 specimens (Tab. 2, Tab. S2). Two of these tumors initially showed *EGFR* amplification and two showed *PDGFRA* amplification, which were, however, not retained in cell lines – only the neoplastic cells without these amplifications were proliferating interminably *in vitro* (Tab. 2, Fig. 1A, Tab. S2). Moreover, one of these tumors originally showed *EGFRvIII* expression, which was also not retained in the cell line (Tab. 2). The complex genetic alterations in the 7 stabilized cell lines were confirmed by different techniques (*i.e.* MLPA, FISH for EGFR, Real-time PCR for *CDKN2A* and *EGFR*, STR and *TP53* sequencing) (Fig. 1).

**Table 2:** Molecular characteristics of all analysed samples in the comparison to the population data and CCLE database data.

	Total	Stable cultures (initial)	Stable cultures (late)	Unstable cultures (initial)	Unstable cultures (late)	Population data	CCLE	
*CDKN2A* deletion	27/56	5/7	5/7	22/49	0/49	42/88	31/40	
*TP53* mutation	13/56	5/7	5/7	8/49	0/49	28/88	27/40	
Both *CDKN2A* deletion and *TP53* mutation	5/56	3/7	3/7	2/49	0/49	5/88	18/40	
*EGFR* amplification	19/56	2/7	0/7	17/49	0/49	23/88	1/40	
*EGFRvIII* expression	15/56	1/7	0/7	14/49	0/49	47/196	2/40*	
*PDGFRA* amplification	7/56	2/7	0/7	5/49	0/49	27/206	1/40**	
		**Stable cultures (initial) *vs.* Unstable cultures (initial)**		**Total *vs.* Population data**		**Stable cultures (late) *vs.* CCLE**		
*CDKN2A* deletion		Χ^2^ = 1.73; p = 0.189		Χ^2^ = 0.00; p = 0.955		Χ^2^ = 0.12; p = 0.726		
*TP53* mutation		**Χ^2^ = 10.43; p = 0.001**		Χ^2^ = 1.24; p = 0.265		Χ^2^ = 0.04; p = 0.837		
Both *CDKN2A* deletion and *TP53* mutation		**Χ^2^ = 11.33; p = 0.001**		Χ^2^ = 0.56; p = 0.455		Χ^2^ = 0.01; p = 0.916		
*EGFR* amplification		Χ^2^ = 0.10; p = 0.749		Χ^2^ = 1.01; p = 0.316		Χ^2^ = 0.17; p = 0.676		
*EGFRvIII* expression		Χ^2^ = 0.92; p = 0.339		Χ^2^ = 0.18; p = 0.667		Χ^2^ = 0.35; p = 0.556		
*PDGFRA* amplification		Χ^2^ = 1.89; p = 0.169		Χ^2^ = 0.01; p = 0.905		Χ^2^ = 0.17; p = 0.667		

The results of the statistical analyses are presented at the bottom.

* The CCLE database do not gathered the data about the *EGFRvIII* occurrence in cell lines, thus the data for *EGFRvIII* expression in cell lines was taken from the literature [24].

** According to CCLE database, the copy number for SW1783 cell line equals 10, therefore we classified this cell line as *PDGFRA*-amplified.

### The Cultures not Providing Stable Cell Lines: Identification of the Overgrowing Cells

**Figure 2:**
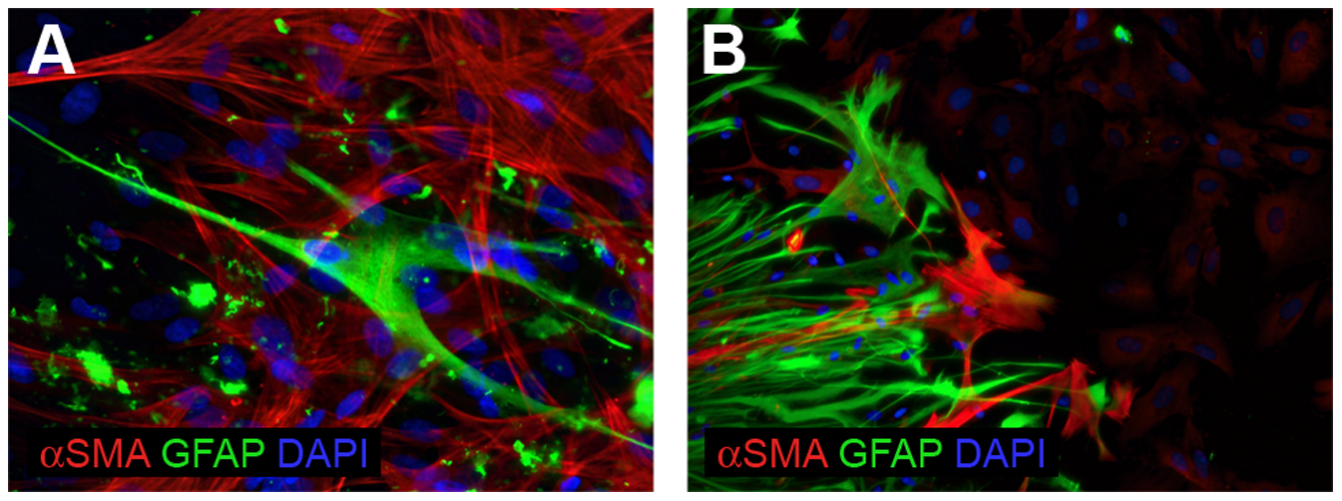
Immunocytochemical identification of the cells overgrowing tumor cells. (A) A late passage of classical monolayer cell culture. The majority of cells expressing αSMA is GFAP(−). A small population of GFAP(+)/α-SMA(−) cells is also visible; (B) The cells invaded radially from the spherical core and further grew as monolayer. Three populations were observed: GFAP-positive; αSMA-positive and double negative.

The molecular analyses revealed that in late passages of cells cultured under the classical conditions virtually only normal cells were present (Tab. 2, Fig. 1C–D). The subpopulation of cells that overgrew glioblastoma cells in these conditions appeared to be the stromal cells, which showed no immunoreactivity for GFAP (a marker of astrocytic cells), but were positive for αSMA (alpha-smooth muscle actin, a protein whose expression was detected for the first time in smooth muscle cells) (Fig. 2A). In addition, a side population of normal cells negative for both markers was also present and remains to be identified (this subpopulation was negative for every analyzed marker listed in Tab. 1). In cultures in which the cells invaded radially from a spherical core and were further cultured as monolayer three subpopulations were detected (GFAP-positive, αSMA-positive and double negative) (Fig. 2B). The stromal cells were passaged up to 15–20^th^ passage when they started to show the features of senescence.

**Figure 3:**
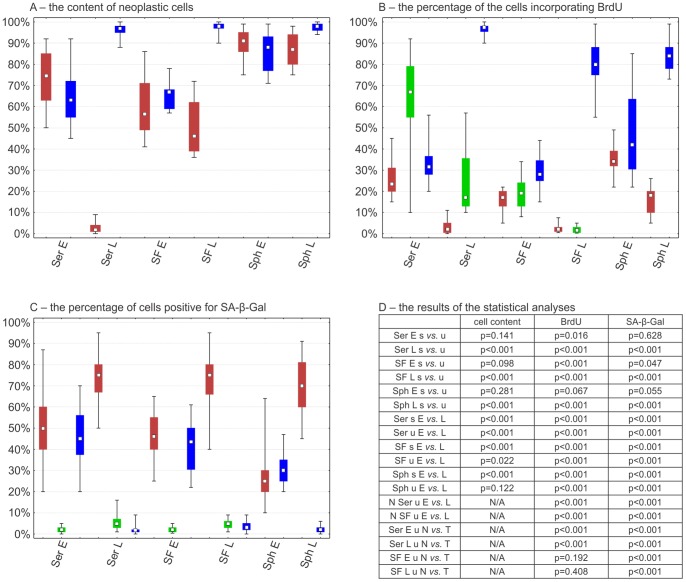
The comparison of cell biology features of glioblastoma cell cultures. The characteristics of cells in different culture conditions are depicted separately: neoplastic cells from cultures which provided a stable cell line (blue bars), neoplastic cells from cultures that did not provide a cell line (red bars) and normal cells from the latter cultures (green bars). (A) The differences in the tumor cell content in glioblastoma cell cultures; (B) The comparison of the percentage of the cells incorporating BrdU during a 7-day incubation; (C) The comparison of the percentage of senescent (positive for SA-β-Gal) cells; (D) The results of Mann-Whitney U test. Ser E – p0-p3 (1–3 weeks) under classical conditions; Ser L – p6-p13 (8–16 weeks) under classical conditions; SF E – p0-p1 (1–3 weeks) under serum-free monolayer conditions; SF L – p3-p5 (6–16 weeks) under serum-free monolayer conditions; Sph E – t0-t2 (up to 3 weeks) of spheroid culturing; Sph L – t4-t8 (3–5 months) of spheroid culturing; E – early passages/transfers; L – late passages/transfers; T – tumor cells, expressing TP53 or EGFR and GFAP; N – normal cells; s – cultures providing stable cell lines, u – cultures not providing stable cell lines.

Different results were obtained with molecular analyses of the monolayer serum-free culture conditions. Albeit having retained the analyzed markers, the cells proliferated slowly and for no longer than 5 passages, which lasted up to 3–4 months. In these conditions both normal and tumor cells were present (Fig. 3A). Conversely, all the analyzed markers have been retained for a minimum of 4 months in spheroids, which consist of neoplastic cells with a marginal subpopulation of normal cells (Fig. 3A).

### The Potential Mechanisms Responsible for the Stabilization Failure

#### Proliferation inhibition

**Figure 4:**
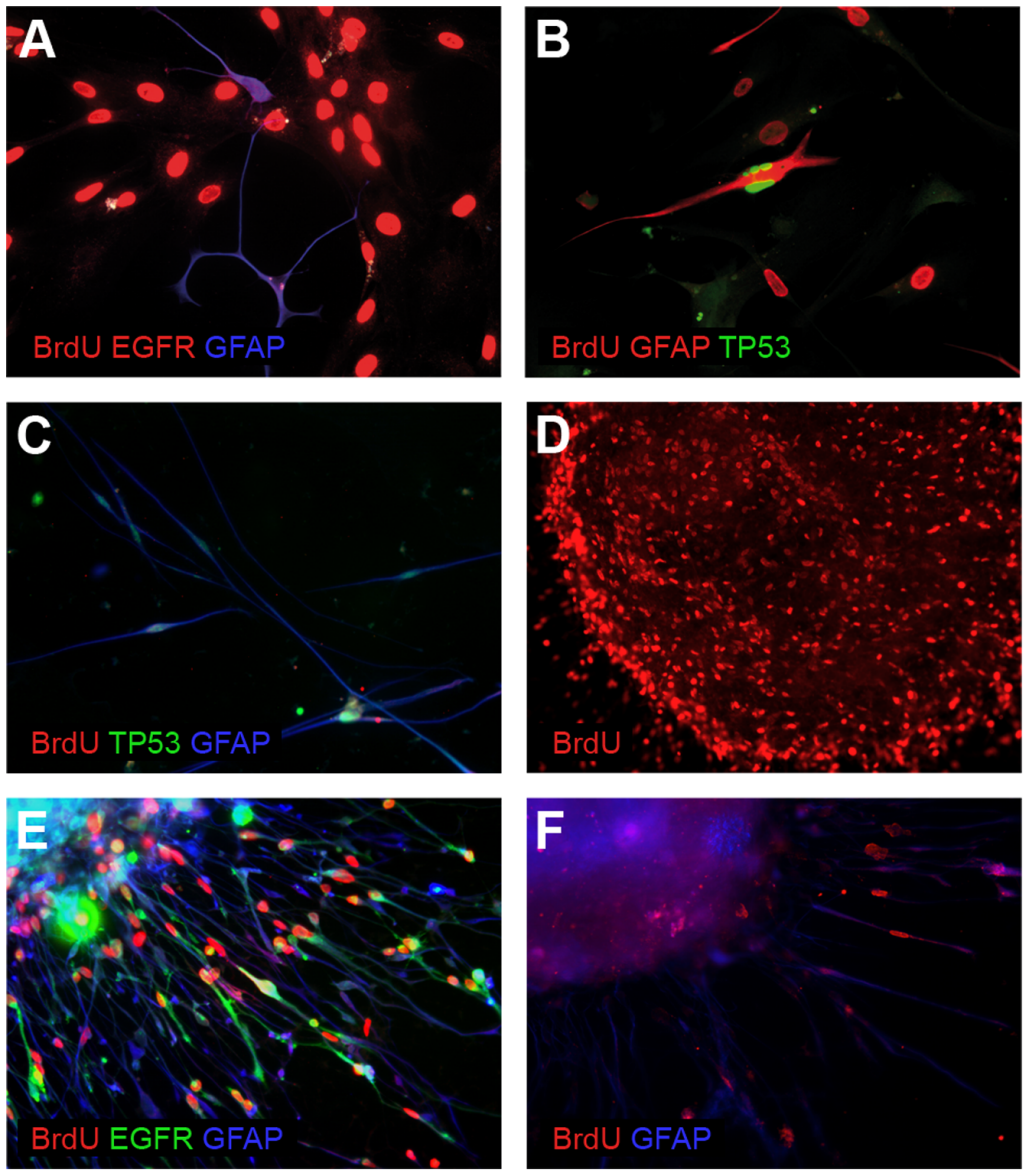
Exemplary results of the immunocytochemical BrdU co-staining in different GB-derived cell cultures. (A,B) An early passage of cells cultured under the classical monolayer conditions after a 7-day incubation with BrdU. In both cases two populations of cells are detectable: (A) BrdU(+)/EGFR(−)/GFAP(−) and BrdU(−)/EGFR(+)/GFAP(+); (B) BrdU(+)/TP53(−)/GFAP(−) and BrdU(−)/TP53(+)/GFAP(+); (C) An early passage of cells cultured under serum-free conditions after 5-day incubation with BrdU, TP53(+)/GFAP(+) tumor cells are negative for BrdU; (D) A spheroid from the 2^nd^ transfer after a 7-day incubation with BrdU, the majority of cells are BrdU-positive; (E) The cells which invaded radially from the spheroid core (2^nd^ transfer) after a 48-hour incubation with BrdU. The majority of EGFR(+)/GFAP(+) cells incorporated BrdU; (F) The cells which invaded from spherical core after 2 months of culturing. Less than 10% of cells incorporated BrdU (10 days of incubation).

The majority of GFAP(+)/TP53(+)/EGFR(+) cells were proliferating (*i.e.* incorporating BrdU during a 7-day incubation) at passage 1 in medium with serum, however, the cells with an inhibited proliferation were also detected at this stage. In late passages the BrdU co-staining showed two populations: BrdU(+)/EGFR(−)/TP53(−)/GFAP(−) and BrdU(−)/EGFR(+)/TP53(+)/GFAP(+) (Fig. 3A–B, 4A–B). On the other hand, the monolayer serum-free conditions moderately favoured the neoplastic cells, nonetheless both cell types quickly became non-proliferative (Fig. 3B, 4C). In contrast, a high proportion of BrdU-incorporating cells was observed even in the 3^rd^ transfer of spheroids (Fig. 3, 4D). The majority of EGFR(+)/TP53(+)/GFAP(+) cells from early spheroid transfers were BrdU-positive (Fig. 4E); after 10 weeks less than 50% of cells incorporated BrdU (Fig. 4F); and finally, tumor cells isolated from glioblastoma became non-proliferative in 3D conditions as well.

#### Senescence

**Figure 5:**
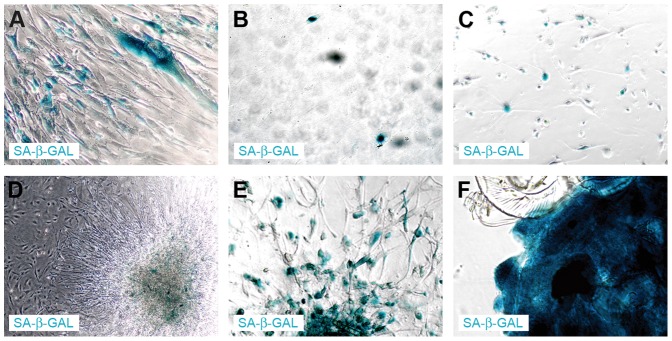
Enzymocytochemical staining showing the senescence of glioblastoma cells cultured under various conditions. SA-β-Gal-positive cells were observed in all tested conditions. (A) An early passage of GB cells under monolayer serum conditions; (B) A late passage of GB cells under monolayer serum conditions; (C) A late passage of GB cells under monolayer serum-free conditions; (D) The cells in an early spheroid; (E) The cells in a late spheroid; (F) The cells in a late floating spheroid.

**Figure 6:**
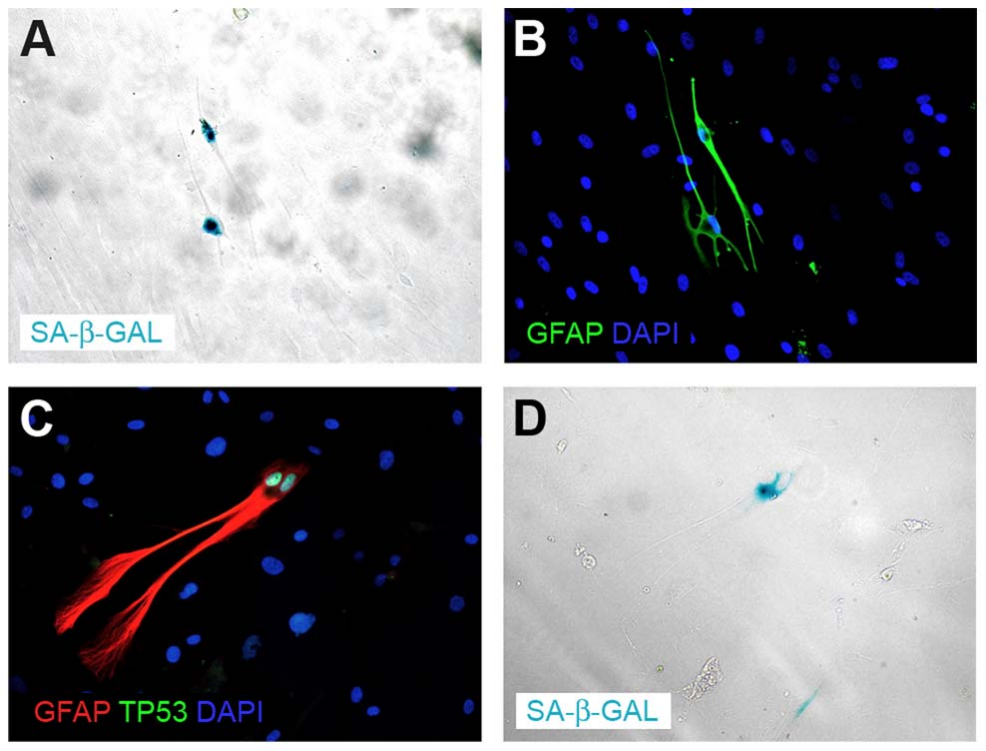
The senescence of neoplastic cells verified by the combination of immunocytochemistry and enzymocytochmistry. (A,B) A late passage of cells cultured under classical conditions. Only GFAP-positive cells show SA-β-Gal activity; (C,D) A late passage of cells cultured under classical conditions. Only TP53(+)/GFAP(+) cell shows SA-β-Gal activity.

**Figure 7:**
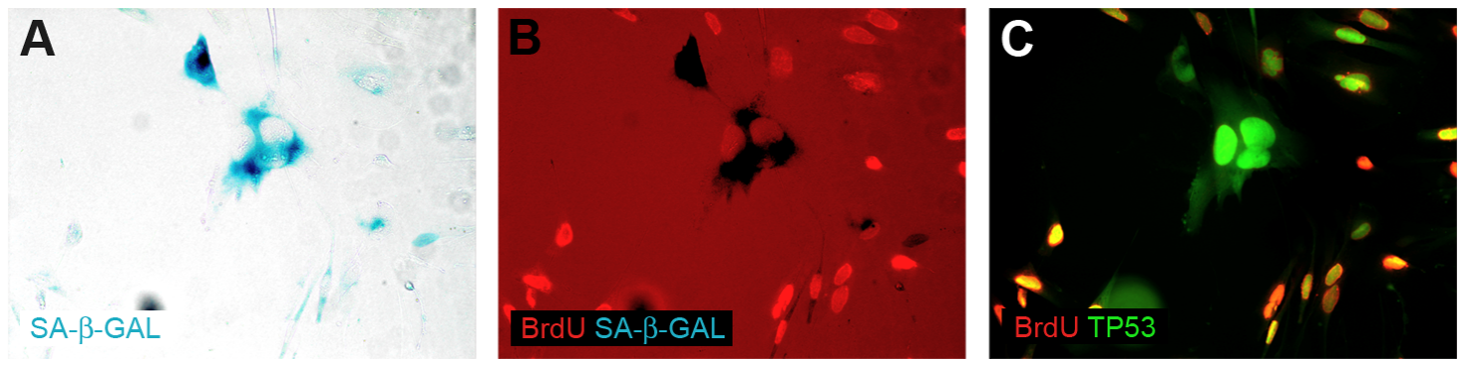
The neoplastic cell senescence verified by two methods (BrdU incorporation assay and SA-β-Gal assay). (A–C) Only two populations of cells are visible: BrdU(−)/SA-β-Gal(+)/TP53(+) and BrdU(+)/SA-β-Gal(−)/TP53(+).

**Figure 8:**
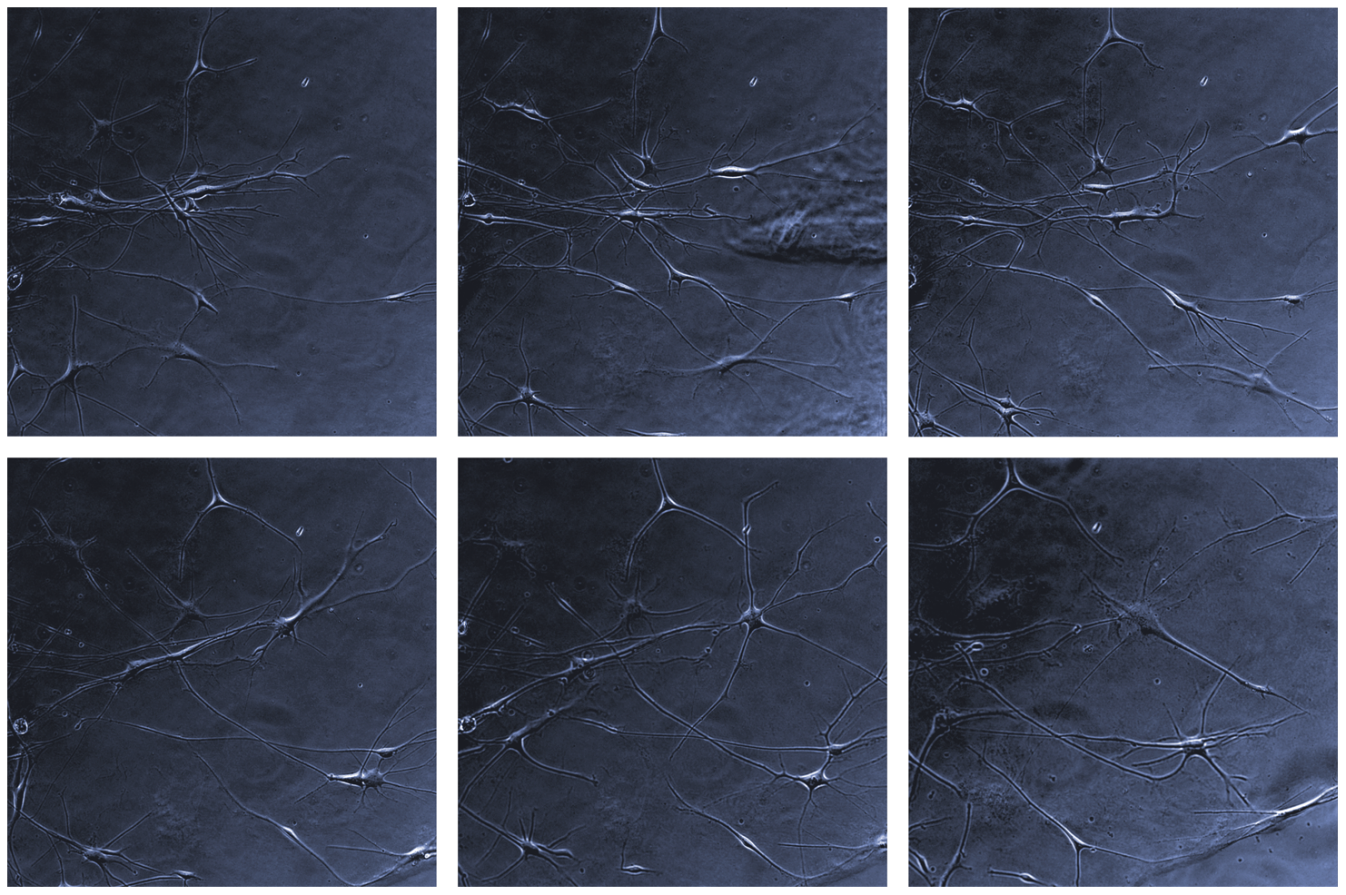
Real-time phase microscopy monitoring of glioblastoma cells. During the 9-day observation (images taken in 36-hour periods) the cells grew and elongated, however, did not proliferate.

The lack of BrdU incorporation was an inspiration to verify if the non-proliferative neoplastic cells are senescent. Intriguingly, we detected the senescent cells in all tested conditions (Fig. 3C, 5). To analyze this issue from a wider perspective we employed a new technical approach based on the combination of enzymocytochemistry with immunocytochemistry and, in further experiments, with BrdU incorporation assay. It allowed us to determine that in the heterogeneous culture (such as primary cultures) in the early passages under the classical conditions, in fact, the GFAP(+)/TP53(+) neoplastic cells were the senescent cells (Fig. 6). In contrast, in late passages only single SA-β-Gal-positive cells were detected, which is consistent with the fact that the neoplastic cells constitute only a minor subpopulation at this phase (Fig. 3B, 5A–B). Normal cells did not show SA-β-Gal activity at these passages (Fig. 3B). Similarly, under monolayer serum-free conditions a high activity of SA-β-Gal was observed (Fig. 3B, 5C). The spheroid cultures postponed the occurrence of senescence (Fig. 3B, 5D–F), however, neither the serum-free 2D nor 3D conditions protected the cultures against the stabilization failure in the majority of cases. These observations were confirmed also with the combination of BrdU incorporation assay with SA-β-Gal assay and immunocytochemistry (Fig. 7) and with real-time phase microscopy observation (Fig. 8).

**Figure 9:**
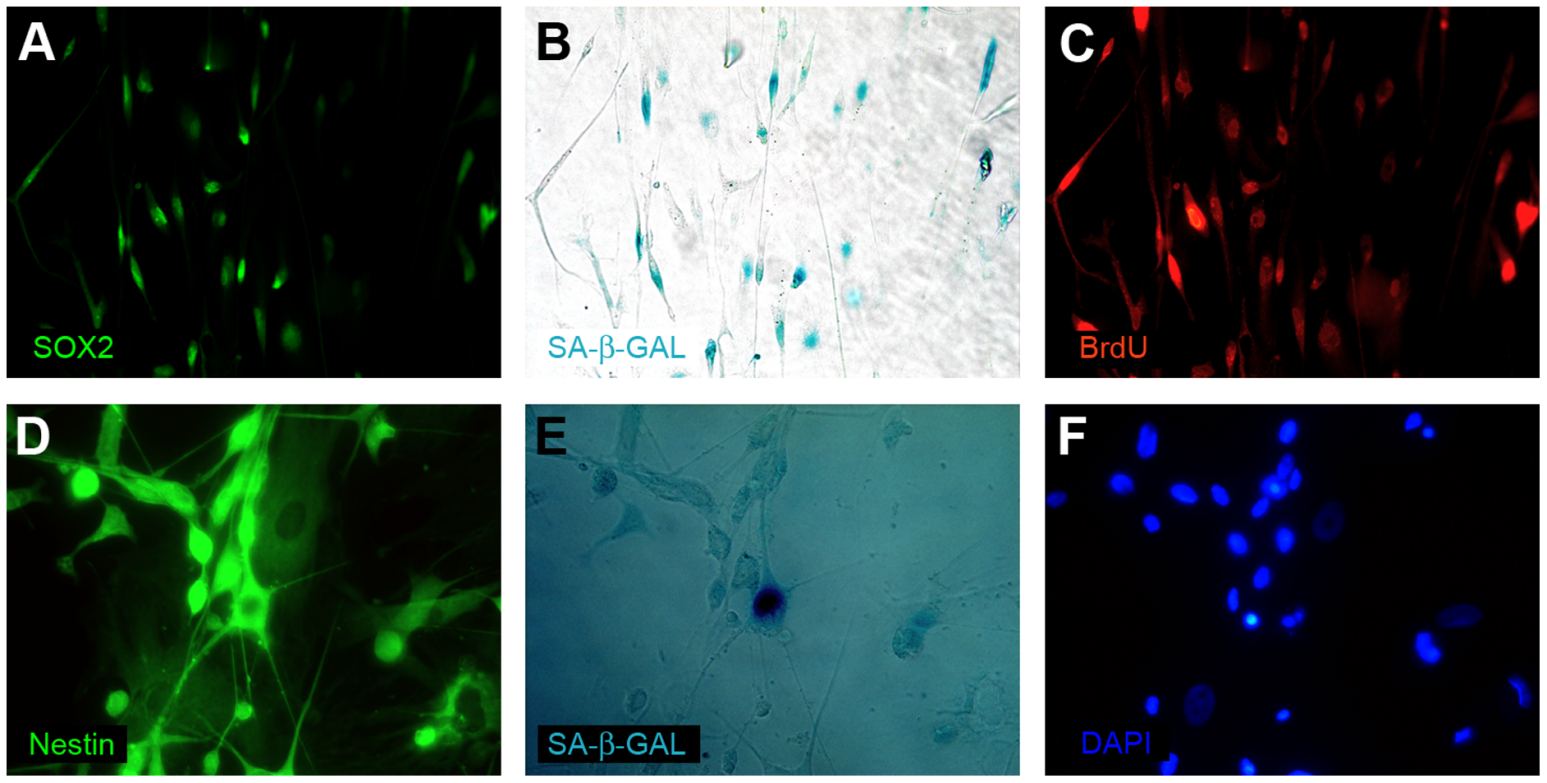
The senescence of glioblastoma cells with stem cells characteristics verified by the combination of immunocytochemistry and enzymocytochemistry. (A,B,C) An early passage of cells cultured under the classical conditions. SOX2-positive cells show SA-β-Gal activity and do not incorporate BrdU; (D, E, F) An early passage of cells cultured under the classical conditions. Nestin-positive cell shows SA-β-Gal activity.

Our analyses of cells with stem markers showed that in all the tested conditions a significant fraction of Nestin-positive and SOX2-positive cells (varying between cases and passages, up to 50%) were negative for BrdU and positive for SA-β-Gal (Fig. 9A, B, C, D, E, F). Therefore, these results imply that a subpopulation of neoplastic cells with the features of stem cells becomes senescent *in vitro*. In addition, we analyzed the cells released from spheroids in order to assess their migratory potential. Nonetheless, none of the observed populations (β-Gal(+)SOX2(+);β-Gal(−)SOX2(+);β-Gal(+)SOX2(−); β-Gal(−)SOX2(−)) was characterized by a longer/shorter distance from the core of the spheroid.

#### Mitotic catastrophe, apoptosis and neosis

**Figure 10:**
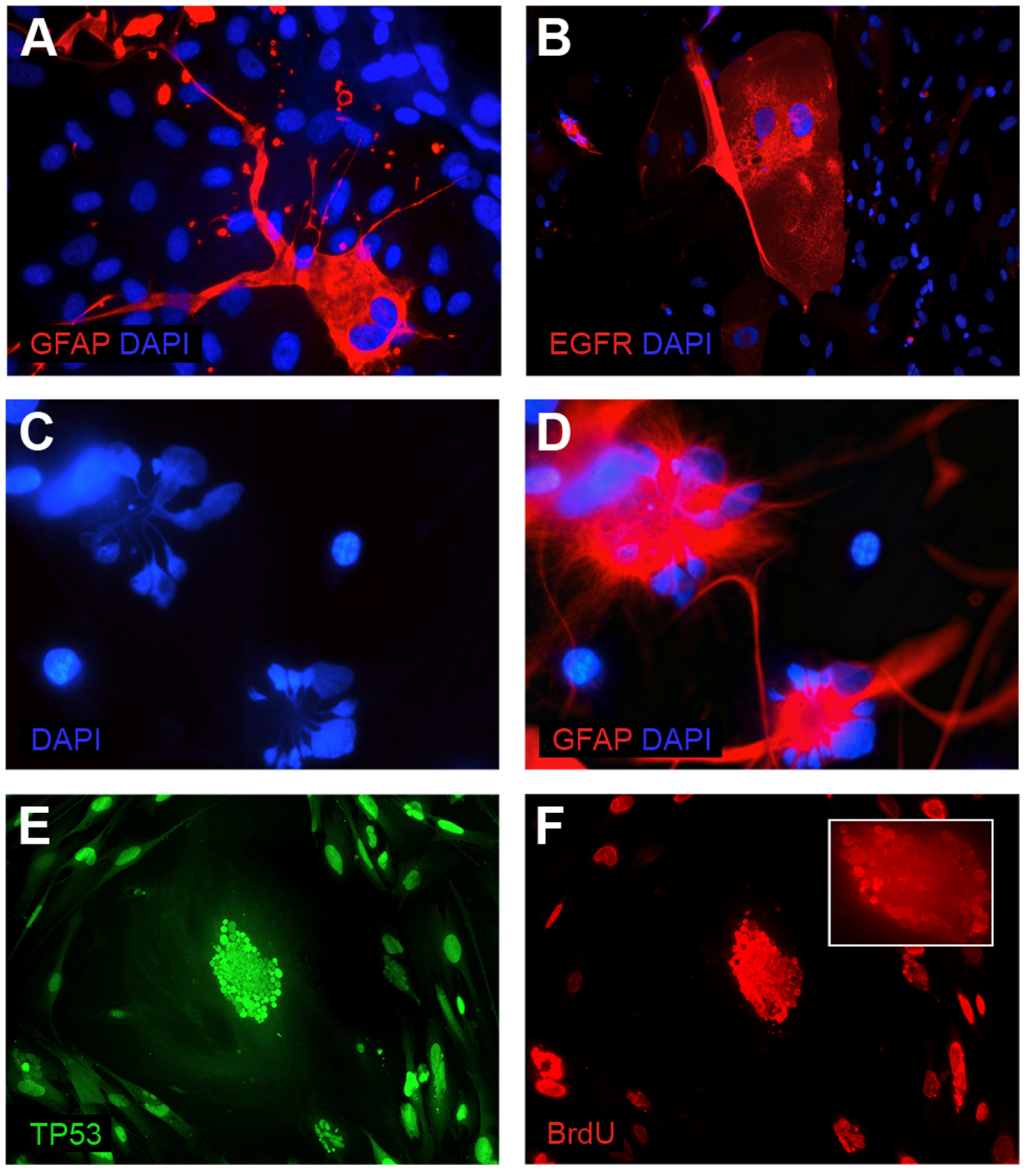
Examples of the additional mechanisms responsible for the glioblastoma cell lines stabilization failure. (A,B) Neoplastic cells with the features of mitotic catastrophe (large planar cells with two nuclei): positive for GFAP (A) and for EGFR (B); (C,D) GFAP-positive cells with the features of apoptosis; (E,F) TP53-positive cell showing the features of neosis; nuclear budding visible, only a portion of the micronuclei is BrdU-positive.

Apart from the senescence, some GB cells showed the features of mitotic catastrophe *in vitro* (Fig. 10A–10B), which was observed in monolayer conditions both with and without serum in all analysed cases. Still, the proportion of the cells showing lack of BrdU incorporation and SA-β-Gal activity was higher than that of cells showing the features of mitotic catastrophe. Although these two phenomena are in general mutually exclusive, several cells showing the features of mitotic catastrophe according to the established criteria were SA-β-Gal-positive. In addition, we also observed apoptotic neoplastic cells and cells described previously as undergoing neosis with a visible nuclear budding (micronucleation) (Fig. 10C, D, E, F) [25], [33]–[35].

#### Invasive potential of glioblastoma cells

**Figure 11:**
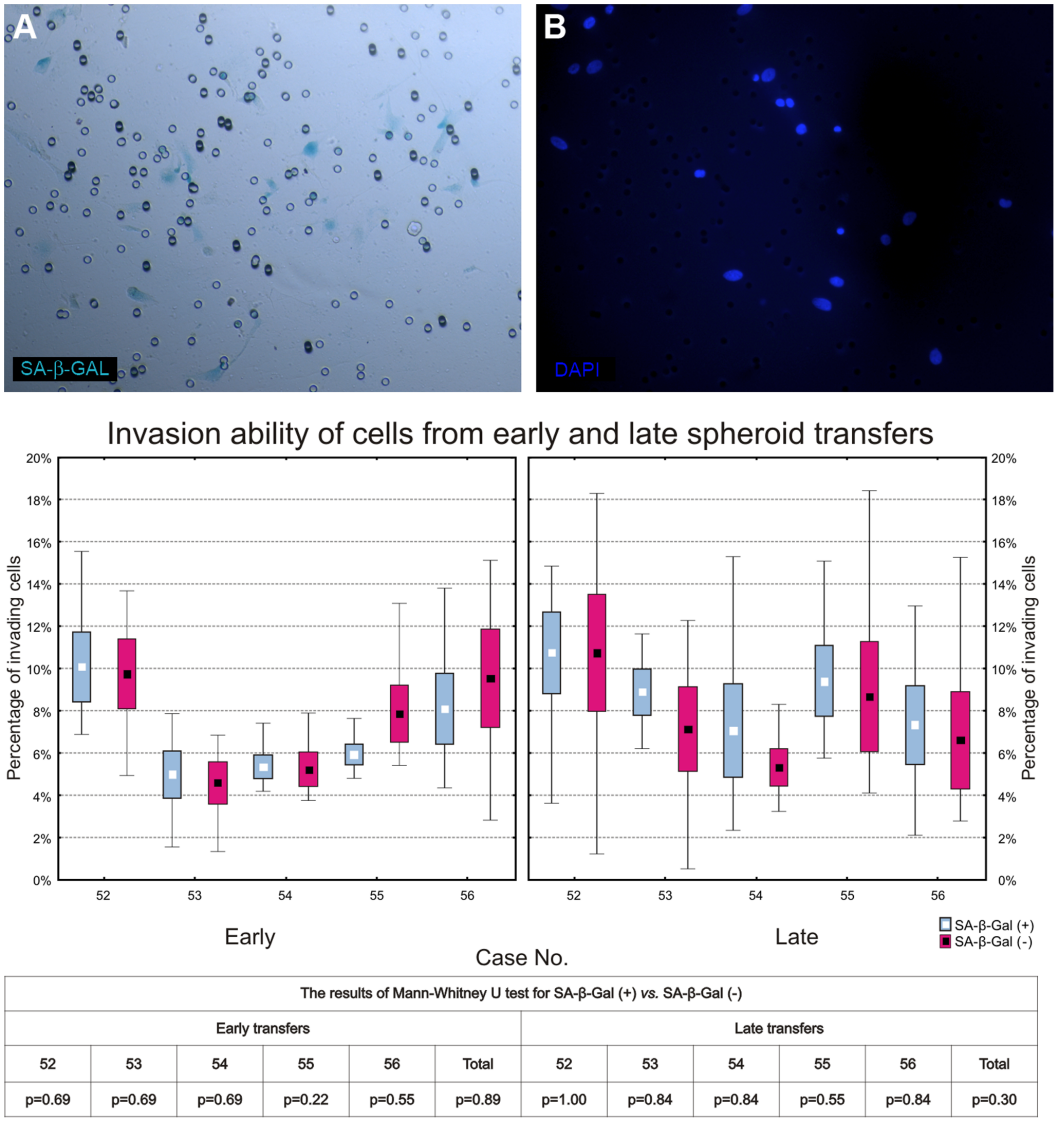
Invasion ability of glioblastoma cells invading from spheroid cultures. (A, B) The GB cells form late transfers of spheroids which invaded thorough the Matrigel-coated filter to the lower surface of the filter of invasion chamber stained for SA-β-Gal activity (A) and with DAPI (B). (C) Box-whisker plots (mean, std. error and min/max values) showing percentage of invading SA-β-Gal (+) and SA-β-Gal (−) cells from early and late transfers of spheroids. The results of the statistical analysis (using Mann-Whitney U test) are presented for each case separately and for the cumulative group in the table below.

Next, we asked whether the functional switch between proliferation and invasion (so-called “go or grow” hypothesis) could explain the observed decrease in proliferation of GB cells *in vitro*. For that purpose we performed the invasion assay in the spheroids cultures (from early and late transfers) from 5 glioblastoma specimens. Neoplastic SA-β-Gal-positive and -negative cells were present in each culture, yet neither was found to be significantly more invasive (Fig. 11).

### Molecular Profile of the Stabilized Cell Lines vs. the Cultures not Providing Stable Cell Lines

We detected the *EGFR* gene amplification in 19 out of 56 cases, screened by Real-Time PCR and confirmed by FISH and MLPA (Tab. 2, Tab. S2). Among the samples initially showing *EGFR* amplification, a cell line was stabilized in 2 cases, however, in both it lost the amplification, but retained *i.a.* LOH, *CDKN2A* deletion and chromosome 7 polysomy. Moreover, one of these two samples showed initially an increased *PDGFRA* gene copy number and the expression of *EGFRvIII*, however, neither was retained in culture.

Next, basing on *TP53* sequencing and *PDGFRA*/*EGFR* amplification results, among all the 56 cases there were 16 marked as Classical-like (CL) and 19 as Proneural-like (PNL), while the stabilized cell lines were obtained from 6 samples marked as PNL and 1 as Other (data in table S2). We tried to verify these observations using the expression data. Nonetheless, an analysis of several selected genes characteristic for specific subtypes as described by Verhaak *et al.* did not allow for an unambiguous classification (the calculated ratios are presented in table S3).

Finally, taking into account that *TP53* mutation and *CDKN2A* deletion are described as mutually exclusive in GB, it was surprising to observe that 3 of the 7 stabilized cell lines had both alterations [28], [29], [36]. Moreover, the analysis of CCLE database revealed that 45% of glioblastoma cell lines have the combination of *TP53* mutation and *CDKN2A* deletion (Tab. 2). Among the specimens that did not provide stable cell lines such combination was uncommon, which is concordant with the population data [28]–[30].

## Discussion

The culturing of glioblastoma cells is troublesome. We were able to stabilize a cell line in 7 of the 56 specimens. In general, an extended culturing under the classical conditions consistently resulted in a gradual loss of neoplastic cells, which were rapidly negatively selected against normal cells (most likely that described by Clavreul *et al*. glioblastoma associated stromal cells, GASCs) [7]. On the other hand, in the monolayer serum-free conditions the prevalence of tumor cells was maintained. Albeit inhibiting the proliferation of normal cells, in most cases these conditions did not stimulate the effective proliferation of neoplastic cells, and thus, did not enhance the stabilization efficiency. In this study, the spheroid structures were the most efficient in retaining the general composition of cells and in stimulating the propagation of tumor cells. Still, albeit postponing the negative processes, such conditions did not eliminate them completely, and thus, did not result in the stabilization of additional cell lines. Therefore, neither the monolayer serum-free conditions nor the spheroids increased the efficiency of cell line stabilization in comparison to the classical conditions, however, they prolonged the retention of neoplastic cells in cultures.

The senescence of GB cells has not been analysed thoroughly thus far, however, the role of this process in carcinogenesis is gaining recognition [37], [38]. A combination of immunocytochemistry, BrdU incorporation assay and enzymocytochemistry was employed to ensure its reliable analysis which proved the senescence of glioblastoma cells. In general, the senescence of neoplastic cells is considered to require a potent inductor (such as conventional chemotherapy, radiation or differentiating agents), therefore, the fact that it may be triggered spontaneously in glioblastoma cells *in vitro* is a crucial observation, which has not been emphasised before [39]. Albeit designed to promote the neoplastic cell survival and proliferation, the *in vitro* conditions may in fact contain factors triggering senescence. In this case it may be regarded as either spontaneous or idiopathic, since no specific inductor has been identified thus far. Moreover, if a glioblastoma specimens containing cells resistant to senescence (*i.e.* able to proliferate infinitely), usually such cells constitute only a minor subpopulation. Clearly, the senescence is responsible for the failure in cell line stabilization to some extent, and therefore, identifying the responsible factors should facilitate developing new culture protocols enabling the stabilization of a wider representation of glioblastoma cells. Nonetheless, the senescence is a dichotomous process playing both a pro- and antineoplastic role *in vivo* [37], [38], [40]. Although the senescent cells do not proliferate, they show a secretory phenotype supporting other subpopulations of the tumor. Therefore, a proper balance between senescent and proliferative cells is required for invasion, metastasis, *etc.* [41]. Moreover, since triggering or inhibiting senescence are regarded as potential novel anticancer approaches, the presence of senescence-resistant cells should be taken into account in the inclusion criteria [42]–[46].

Additionally, in this study among the cells which become senescent without any additional trigger we report glioblastoma cells expressing stem cell markers. It is a crucial observation, which indicates a link between the decrease in the glioma cell growth and senescence. On the other hand, we are aware that the definition of glioma stem cells is elusive and the possibility that a large proportion of GB cells has the possibility to play the stem cell role.

Consequently, the presented data lead to the question of the role of senescent cells in glioblastoma. One of the possibilities is the hypothesis saying that cells cannot simultaneously express proliferative and infiltrating phenotype, therefore, the senescence would be linked to the invasion. The analysis of cell lines showed that usually the more proliferative cell lines are less invasive and, analogously, the more invasive cell lines are less proliferative [47], [48]. To date, there are several reports either opposing or supporting the “go or grow” hypothesis [49], [50]. The senescent cells, which are by definition non-proliferative, were not subjected to such analyses. Furthermore, senescent cells tend to grow to a very large size (with the length up to 2000 µm and cell body diameter without filopodia up to 250–400 µm) (Fig. 8). For such cells the process of migration/invasion is extremely difficult. Our data do not support the link between senescence and invasiveness, and we suggest that other potential roles of the senescent cells should be investigated as well. Another possibility may be inferred from the fact that glial tumors contain a large proportion of infiltrating normal cells (including GASCs, endothelial cells, microglia, *etc.*), which are strongly influenced by the factors released by GB cells. Taking into account their secretory phenotype, senescent cells may constitute an acting element of a neoplastic paracrine loop/system. Nonetheless, such a hypothesis requires a careful verification.

The senescence was not the only observed mechanism responsible for GB cell line stabilization failure. Mitotic catastrophe also played its part. Mostly, these mechanisms were described as resulting from the applied chemotherapeutics or irradiation [25], [39], [51]. Nevertheless, in all the tested conditions the proportion of senescent neoplastic cells was higher than that of cells with the features of mitotic catastrophe or apoptosis.

Different classifications of glioblastoma into molecular subtypes have been performed by several authors, the most recognized of which was the one proposed by Verhaak *et al.* and Noushmehr *et al.* [22], [23]. Le Mercier *et al.* proposed a simplified approach, based on the IHC analysis of *TP53*, *EGFR* and *PDGFRA*, to identify the Classical-like and Proneural-like subtypes [21]. We used our results of the genomic analyses of these genes for an analogous classification (Tab. S2). Intriguingly, we observed a significant overrepresentation of the PNL subtype among the stable cell lines and none with the CL subtype. To verify these observations we analyzed the expression of several genes characteristic for specific subtypes (ratios in table S3). Nonetheless, we were not able to reliably infer the subtypes from the expression results.

Importantly, we observed that 45% of the stabilized cell lines (our data and CCLE) show both *TP53* mutation and homozygous deletion of *CDKN2A*, while these alterations are described as mutually exclusive in glioblastoma *in vivo* [28]–[30], [36]. Indeed, among the 49 specimens which did not provide stable cell lines there were only 2 such cases. Notably, nearly a half of glioma cell lines represents a small group of tumors with a combination of specific genetic alterations, which may possibly be related to their insusceptibility to spontaneous *in vitro* senescence. This observation may be crucial for the identification of the responsible mechanisms.

### Conclusions

To summarize, in spite of the use of different cell culture conditions, still, the majority of GB specimens do not provide stable cells lines. The identification of the reasons for their negative selection under classical culture conditions and the longer survival in 3D spheroids may help in refining GB cell culture protocols and widen the knowledge of glioblastoma cell biology. Glioblastoma cells present *in vitro* several mechanisms responsible for the failure in cell line stabilization: primarily senescence, and, to a lesser extent, mitotic catastrophe and apoptosis. Three-dimensional conditions prolong the survival of GB cells *in vitro*, but even in such conditions the cells become non-proliferative and show the features of senescence. Intriguingly, our data show that the observed senescence may depend on *TP53* and *CDKN2A* (Tab. 2).

To date, the obstacles in glioblastoma cell culturing are recognized as a problem due to the lack of the appropriate *in vitro* representation, which is particularly important from the drug testing perspective. On the other hand, such obstacles may potentially be transformed into a novel antineoplastic therapy, which could be nontoxic for normal cells.

## Supporting Information

Table S1 **Primer sequences.**
(DOCX)

Table S2 **Detailed molecular characteristics of all analysed samples.** W/T – wild type; del – whole gene deletion; PNL – Proneural like; CL – Classical-like. Data for stabilized cell lines were applicable.
(DOCX)

Table S3 **Results of the expression analysis of the selected genes.**
(DOCX)
