# Supplementary material for: The Failure in the Stabilization of Glioblastoma-Derived Cell Lines: Spontaneous In Vitro Senescence as the Main Culprit
Source: PLoS One. 2014 Jan 30;9(1):e87136. doi: 10.1371/journal.pone.0087136 (PMC3910690; doi:10.1371/journal.pone.0087136)
Supplement: Table S1 — Primer sequences. (DOCX) [file pone.0087136.s001.docx]

**Table S1.** Primer sequences.

| **Gene** | **Sense primer** | **Antisense primer** |
| --- | --- | --- |
| *RNaseP* | 5'-GGGAGATGCGGAAGAATGT-3' | 5'-CCTCCAGTCAGCCACAGAA-3' |
| *EGFR (amplification)* | 5'-CACACCCCTGACTCTCCACT-3' | 5'-GAGACAATCCTGTGAGCTTGG-3' |
| *GPER* | 5'-CATCTGGACGGCAGGTAAGT-3' | 5'-CCCTCAGCCGGTAGTTTTC-3' |
| *CDKN2A (exon 1)* | 5’-CAACGCACCGAATAGTTACG-3’ | 5’-CTGCAAACTTCGTCCTCCAG-3’ |
| *CDKN2A (exon 2)* | 5’-ACCAGAGGCAGTAACCATGC-3’ | 5’-TGGAAGCTCTCAGGGTACAAA-3’ |
| *TP53 (amplification)* | 5’-GTGCAGCTGTGGGTTGATT-3’ | 5’-GCAGTGCTCGCTTAGTGCTC-3’ |
| *TP53 (sequencing)* | 5’-GCCATCTACAAGCAGTCACA-3’ | 5’-CCCTTTCTTGCGGAGATTCT-3’ |
| *GUSB* | 5'-CTCATTTGGAATTTTGCCGATT-3' | 5'-CCGAGTGAAGATCCCCTTTTTA-3' |
| *EGFRvIII* | 5'-GGCTCTGGAGGAAAAGAAAGGTAAT-3' | 5'-TCCTCCATCTCATAGCTGTCG-3' |
| *EGFR (expression)* | 5'-CGGGCTCTGGAGGAAAAGAA-3' | 5'-ACATCCTCTGGAGGCTGAGA-3' |
| *NF1* | 5'-GGTGGAATGGGTCCAGGC-3' | 5'-GACATTCCTTGTTGTGCTCAGT-3' |
| *TP53 (expression)* | 5'-AGAGCTGAATGAGGCCTTGG-3' | 5'-TATGGCGGGAGGTAGACTGA-3' |
| *PDGFB* | 5'-CTCGTGGAAGAAGGAGCCTG-3' | 5'-GGAGATCTCGAACACCTCGG-3' |
| *CHI3L1* | 5'-CCAGTGCTGCTCTGCATAC-3' | 5'-GGTCAAGGGCATCTGGGAAG-3' |
| *MGMT* | 5'-GTGGGAGGAGCAATGAGAGG-3' | 5'-CAGTCCTCCGGAGTAGTTGC-3' |
| *XRCC1* | 5'-AAATTGTTTGCAGCCAGCCC-3' | 5'-GGCCTCTGCCTCATCTTTGT-3' |
| *GABRA1* | 5'-TGTCCGATGCATTTGGAGGA-3' | 5'-GCTGGCTCTCTGGTCCATTC-3' |
| *HES1* | 5'-ATGACAGTGAAGCACCTCCG-3' | 5'-CGTTCATGCACTCGCTGAAG-3' |
