# Supplementary material for: The Failure in the Stabilization of Glioblastoma-Derived Cell Lines: Spontaneous In Vitro Senescence as the Main Culprit
Source: PLoS One. 2014 Jan 30;9(1):e87136. doi: 10.1371/journal.pone.0087136 (PMC3910690; doi:10.1371/journal.pone.0087136)
Supplement: Table S2 — Detailed molecular characteristics of all analysed samples. W/T – wild type; del – whole gene deletion; PNL – Proneural like; CL – Classical-like. Data for stabilized cell lines were applicable. (DOCX) [file pone.0087136.s002.docx]

**Table S2.** Detailed molecular characteristics of all analysed samples. W/T – wild type; del – whole gene deletion; PNL – Proneural like; CL – Classical-like. Data for stabilized cell lines were applicable.

| Case # | Stabilized / unstabilized | *CDKN2A* | | *TP53* | | | *EGFR* gene copy number | | | *EGFRvIII* expression | | | *PDGFR* gene copy number | | | Subtype | |
| --- | --- | --- | --- | --- | --- | --- | --- | --- | --- | --- | --- | --- | --- | --- | --- | --- | --- |
|  |  | frozen sample | cell line | frozen sample | | cell line | frozen sample | | cell line | frozen sample | | cell line | frozen sample | | cell line | frozen sample | cell line |
| 1 | stabilized cell lines | del | del | mutated | | mutated | polisomy | | polisomy | no | | no | increased | | increased | PNL | PNL |
| 2 |  | del | del | mutated | | mutated | polisomy | | polisomy | no | | no | normal | | normal | PNL | PNL |
| 3 |  | del | del | W/T | | W/T | normal | | normal | no | | no | normal | | normal | other | other |
| 4 |  | W/T | W/T | mutated | | mutated | polisomy | | polisomy | no | | no | normal | | normal | PNL | PNL |
| 5 |  | del | del | mutated | | mutated | amplification + polisomy | | polisomy | no | | no | normal | | normal | PNL | PNL |
| 6 |  | del | del | W/T | | W/T | amplification + polisomy | | polisomy | yes | | no | increased | | normal | PNL | other |
| 7 |  | W/T | W/T | mutated | | mutated | normal | | normal | no | | no | normal | | normal | PNL | PNL |
| 8 | unstabilized cell cultures | W/T | | W/T | | | amplification | | | yes | | | normal | | | CL | |
| 9 |  | W/T | | mutated | | | normal | | | no | | | normal | | | PNL | |
| 10 |  | W/T | | W/T | | | amplification | | | no | | | normal | | | CL | |
| 11 |  | W/T | | W/T | | | amplification | | | yes | | | normal | | | CL | |
| 12 |  | W/T | | mutated | | | normal | | | yes | | | normal | | | PNL | |
| 13 |  | W/T | | W/T | | | amplification | | | no | | | normal | | | CL | |
| 14 |  | W/T | | W/T | | | amplification | | | no | | | normal | | | CL | |
| 15 |  | del | | mutated | | | normal | | | no | | | normal | | | PNL | |
| 16 |  | W/T | | W/T | | | normal | | | no | | | normal | | | other | |
| 17 |  | W/T | | mutated | | | normal | | | no | | | normal | | | PNL | |
| 18 |  | del | | W/T | | | normal | | | no | | | normal | | | other | |
| 19 |  | del | | W/T | | | amplification | | | no | | | normal | | | CL | |
| 20 |  | del | | W/T | | | normal | | | no | | | normal | | | other | |
| 21 |  | W/T | | W/T | | | normal | | | yes | | | normal | | | other | |
| 22 |  | del | | W/T | | | normal | | | no | | | normal | | | other | |
| 23 |  | del | | W/T | | | polisomy | | | no | | | normal | | | other | |
| 24 |  | W/T | | W/T | | | amplification + polisomy | | | no | | | increased | | | CL | |
| 25 |  | del | | W/T | | | normal | | | no | | | normal | | | other | |
| 26 |  | del | | W/T | | | polisomy | | | no | | | increased | | | other | |
| 27 |  | W/T | | mutated | | | normal | | | yes | | | normal | | | PNL | |
| 28 |  | del | | W/T | | | normal | | | no | | | normal | | | other | |
| 29 |  | del | | W/T | | | normal | | | yes | | | normal | | | other | |
| 30 |  | del | | W/T | | | amplification | | | yes | | | normal | | | CL | |
| 31 |  | W/T | | W/T | | | normal | | | no | | | increased | | | PNL | |
| 32 |  | del | | W/T | | | normal | | | no | | | normal | | | other | |
| 33 |  | W/T | | W/T | | | normal | | | no | | | normal | | | other | |
| 34 |  | W/T | | W/T | | | polisomy | | | no | | | normal | | | other | |
| 35 |  | W/T | | W/T | | | amplification | | | no | | | normal | | | CL | |
| 36 |  | del | | W/T | | | amplification + polisomy | | | yes | | | normal | | | CL | |
| 37 |  | del | | mutated | | | polisomy | | | no | | | normal | | | PNL | |
| 38 |  | W/T | | W/T | | | normal | | | no | | | normal | | | other | |
| 39 |  | W/T | | W/T | | | normal | | | yes | | | normal | | | other | |
| 40 |  | del | | W/T | | | normal | | | no | | | normal | | | other | |
| 41 |  | del | | W/T | | | amplification | | | no | | | normal | | | CL | |
| 42 |  | del | | W/T | | | normal | | | no | | | normal | | | other | |
| 43 |  | W/T | | mutated | | | normal | | | no | | | normal | | | PNL | |
| 44 |  | W/T | | W/T | | | amplification + polisomy | | | yes | | | normal | | | CL | |
| 45 |  | W/T | | W/T | | | normal | | | no | | | normal | | | other | |
| 46 |  | del | | W/T | | | amplification | | | yes | | | normal | | | CL | |
| 47 |  | del | | W/T | | | amplification + polisomy | | | yes | | | normal | | | CL | |
| 48 |  | del | | W/T | | | amplification + polisomy | | | yes | | | normal | | | CL | |
| 49 |  | W/T | | W/T | | | normal | | | no | | | increased | | | PNL | |
| 50 |  | del | | W/T | | | polisomy | | | no | | | normal | | | other | |
| 51 |  | W/T | | W/T | | | normal | | | no | | | normal | | | other | |
| 52 |  | W/T | | W/T | | | normal | | | no | | | increased | | | PNL | |
| 53 |  | del | | W/T | | | normal | | | no | | | normal | | | other | |
| 54 |  | W/T | | mutated | | | normal | | | no | | | normal | | | PNL | |
| 55 |  | W/T | | W/T | | | amplification | | | yes | | | normal | | | CL | |
| 56 |  | W/T | | W/T | | | amplification | | | yes | | | normal | | | CL | |
| n=56 |  |  |  |  |  | |  |  | |  |  | |  |  | |  | |
