# Supplementary material for: The Failure in the Stabilization of Glioblastoma-Derived Cell Lines: Spontaneous In Vitro Senescence as the Main Culprit
Source: PLoS One. 2014 Jan 30;9(1):e87136. doi: 10.1371/journal.pone.0087136 (PMC3910690; doi:10.1371/journal.pone.0087136)
Supplement: Table S3 — Results of the expression analysis of the selected genes. (DOCX) [file pone.0087136.s003.docx]

**Table S3.** Results of the expression analysis of the selected genes.

| Case # | Stabilized / unstabilized | *EGFR* | *NF1* | *TP53* | *CHI3L1* | *PDGFB* | *GABRA1* | *MGMT* | *XRCC1* | *HES1* |
| --- | --- | --- | --- | --- | --- | --- | --- | --- | --- | --- |
| 1 | stabilized cell lines | 0.03 | 1.75 | 0.67 | 0.12 | 0.41 | 11.10 | 2.15 | 0.62 | 0.67 |
| 2 |  | 0.01 | 1.45 | 0.32 | 0.13 | 0.62 | 0.74 | 3.64 | 0.53 | 0.28 |
| 3 |  | 0.02 | 0.61 | 0.87 | 0.40 | 0.11 | 0.00 | 2.91 | 0.26 | 0.39 |
| 5 |  | 0.05 | 2.08 | 0.61 | 0.20 | 1.20 | 29.91 | 2.27 | 0.92 | 0.82 |
| 6 |  | 0.01 | 0.44 | 0.17 | 2.80 | 0.68 | 0.00 | 3.57 | 0.23 | 0.73 |
| 7 |  | 0.02 | 0.98 | 0.95 | 1.15 | 0.18 | 0.00 | 17.90 | 0.40 | 0.58 |
| 14 | unstabilized cell cultures | 0.06 | 1.71 | 0.48 | 0.30 | 0.41 | 0.00 | 7.51 | 0.38 | 0.74 |
| 15 |  | 0.02 | 0.81 | 0.66 | 0.23 | 0.03 | 3.90 | 1.45 | 0.27 | 0.62 |
| 16 |  | 0.01 | 1.03 | 0.37 | 0.02 | 0.34 | 0.00 | 4.88 | 0.93 | 0.53 |
| 19 |  | 0.01 | 0.86 | 0.45 | 0.02 | 0.09 | 0.00 | 3.60 | 0.40 | 0.22 |
| 23 |  | 0.01 | 0.58 | 0.65 | 0.03 | 0.01 | 0.00 | 2.21 | 0.25 | 0.32 |
| 25 |  | 0.01 | 0.26 | 0.41 | 0.83 | 0.14 | 0.54 | 0.31 | 0.21 | 0.38 |
| 26 |  | 0.00 | 0.56 | 0.49 | 0.03 | 0.10 | 0.62 | 2.24 | 0.32 | 0.24 |
| 27 |  | 0.23 | 8.80 | 3.46 | 0.53 | 7.64 | 125.53 | 9.50 | 5.34 | 3.30 |
| 28 |  | 0.01 | 0.15 | 0.26 | 0.20 | 0.21 | 1.54 | 1.58 | 0.22 | 0.36 |
| 29 |  | 0.02 | 0.72 | 0.55 | 1.89 | 0.10 | 0.00 | 0.52 | 0.33 | 1.04 |
| 30 |  | 0.11 | 1.11 | 0.62 | 1.69 | 4.14 | 5.35 | 3.84 | 1.00 | 0.87 |
| 24 |  | 0.63 | 1.66 | 1.06 | 8.17 | 1.22 | 7.42 | 2.60 | 0.63 | 0.84 |
| 35 |  | 0.26 | 1.29 | 0.63 | 6.90 | 0.74 | 16.70 | 3.36 | 0.70 | 1.22 |
| 37 |  | 0.07 | 2.16 | 1.74 | 1.30 | 3.03 | 18.89 | 3.44 | 1.85 | 1.72 |
| 40 |  | 0.07 | 2.39 | 0.68 | 0.10 | 6.02 | 188.49 | 18.34 | 3.17 | 2.43 |
| 41 |  | 0.12 | 1.86 | 0.60 | 2.84 | 1.25 | 0.00 | 7.59 | 1.33 | 1.84 |
| 43 |  | 0.01 | 1.32 | 1.10 | 0.00 | 0.28 | 0.62 | 0.00 | 0.36 | 0.35 |
| 44 |  | 0.79 | 1.79 | 0.76 | 0.31 | 4.13 | 98.90 | 17.65 | 1.79 | 1.49 |
| 45 |  | 0.02 | 0.54 | 0.40 | 1.21 | 1.23 | 62.53 | 4.86 | 0.73 | 0.90 |
| 46 |  | 0.62 | 1.60 | 1.27 | 6.76 | 0.98 | 8.68 | 2.78 | 0.64 | 1.01 |
| 47 |  | 0.57 | 0.52 | 0.37 | 0.31 | 1.71 | 10.14 | 3.03 | 1.52 | 0.95 |
| 48 |  | 0.29 | 0.72 | 0.38 | 1.86 | 0.15 | 2.08 | 0.99 | 0.37 | 0.61 |
| 49 |  | 0.00 | 1.93 | 0.79 | 0.00 | 0.35 | 191.69 | 4.73 | 1.92 | 0.10 |
| 53 |  | 0.23 | 1.07 | 0.59 | 1.34 | 0.97 | 30.50 | 1.86 | 0.50 | 0.65 |
| 55 |  | 0.00 | 0.74 | 1.02 | 0.01 | 1.29 | 0.78 | 2.62 | 1.11 | 0.37 |
| 56 |  | 0.07 | 1.59 | 0.41 | 0.64 | 1.45 | 9.81 | 1.00 | 0.97 | 1.05 |
| n=33 |  |  |  |  |  |  |  |  |  |  |
